# Deep Multi-Modal Transfer Learning for Augmented Patient Acuity Assessment in the Intelligent ICU

**DOI:** 10.3389/fdgth.2021.640685

**Published:** 2021-02-22

**Authors:** Benjamin Shickel, Anis Davoudi, Tezcan Ozrazgat-Baslanti, Matthew Ruppert, Azra Bihorac, Parisa Rashidi

**Affiliations:** ^1^Department of Computer and Information Science and Engineering, University of Florida, Gainesville, FL, United States; ^2^Precision and Intelligent Systems in Medicine (PRISMAP), University of Florida, Gainesville, FL, United States; ^3^Department of Biomedical Engineering, University of Florida, Gainesville, FL, United States; ^4^Department of Medicine, University of Florida, Gainesville, FL, United States

**Keywords:** machine learning, deep learning, transfer learning, intensive care unit, electronic health records, intelligent ICU

## Abstract

Accurate prediction and monitoring of patient health in the intensive care unit can inform shared decisions regarding appropriateness of care delivery, risk-reduction strategies, and intensive care resource use. Traditionally, algorithmic solutions for patient outcome prediction rely solely on data available from electronic health records (EHR). In this pilot study, we explore the benefits of augmenting existing EHR data with novel measurements from wrist-worn activity sensors as part of a clinical environment known as the Intelligent ICU. We implemented temporal deep learning models based on two distinct sources of patient data: (1) routinely measured vital signs from electronic health records, and (2) activity data collected from wearable sensors. As a proxy for illness severity, our models predicted whether patients leaving the intensive care unit would be successfully or unsuccessfully discharged from the hospital. We overcome the challenge of small sample size in our prospective cohort by applying deep transfer learning using EHR data from a much larger cohort of traditional ICU patients. Our experiments quantify added utility of non-traditional measurements for predicting patient health, especially when applying a transfer learning procedure to small novel Intelligent ICU cohorts of critically ill patients.

## 1. Introduction

Patients admitted to a hospital's intensive care unit (ICU) have life-threatening conditions or the propensity to develop them at any moment. An estimated 5.7 million adults are admitted to ICUs in the United States annually, and their precarious and often rapidly-changing state of health necessitates increased monitoring and hospital resources that costs the U.S. healthcare system more than 67 billion dollars every year (1).

A typical ICU stay occurs in an environment of high-frequency patient monitoring involving a wide variety of physiological measurements such as vital sign tracking, bedside nursing assessments, and laboratory test results. These clinical data points serve as a window into patient illness severity, and taken over time can indicate improving or worsening physiological health. The robust clinical data generated during an ICU stay can aid caregivers in diagnosis and influence clinical decision-making regarding medication administration, appropriateness of clinical procedures and surgery, and duration and resource requirement of intensive care.

The rich data associated with a typical ICU stay is routinely captured in modern electronic health record (EHR) systems. As of 2017, more than 99% of U.S. hospitals use some form of EHR (2). These longitudinal systems store a large magnitude of patient information including demographics and admission information, vital signs, diagnoses and procedures, laboratory tests, prescriptions and medications, bedside assessments, clinical notes, and more. While inherently useful for care delivery and administrative hospital tasks like billing, EHR systems also function as a rich source for more automated data-driven patient monitoring applications.

Given the potential for health instability commonly associated with patients undergoing intensive care, the timely and accurate assessment of illness severity is invaluable and can inform shared decision-making among patients, families, and providers. Traditionally, overall patient acuity can be measured using a variety of manual, threshold-based scoring systems such as Sequential Organ Failure Assessment (SOFA) (3), Acute Physiology And Chronic Health Evaluation (APACHE) (4), Simplified Acute Physiology Score (SAPS) (5, 6), Modified Early Warning Score (MEWS) (7), and others. More recently, clinical informatics research has demonstrated the validity and accuracy of more automated machine learning approaches using the rich data from EHR systems (8–12). In particular, modern algorithmic techniques using deep learning have been shown to outperform traditional bedside severity scores for predicting in-hospital mortality as a proxy for real-time patient acuity (13). Automated approaches for assessing patient illness severity can help eliminate reliance on overburdened providers, improve the precision of personalized acuity estimates, and be computed in real-time when combined with streaming EHR platforms.

One potential disadvantage of automated patient monitoring solutions is that such systems are limited to physiological data that is recorded in EHR databases. This common paradigm omits important aspects of patient care, including environmental factors (such as noise, light, and sleep), facial expressions that can indicate pain, agitation, or affective state, and aspects of patient mobility and functional status.

Currently, patient pain can be measured by scoring systems such as the Non-Verbal Pain Scale (NVPS) (14) and the Defense and Veterans Pain Rating Scale (DVPRS) (15), and patient activity can be assessed by scoring systems such as the Progressive Upright Mobility Protocol (PUMP) Plus (16) and the ICU Mobility Scale (IMS) (17). However, these manual scores are much less granular than the corresponding physiological measurements and require either self-reporting or repetitive observations by ICU staff (18, 19). The reduced frequency and granularity of these types of patient data can hinder timely intervention strategies (20–25).

**Figure 1:**
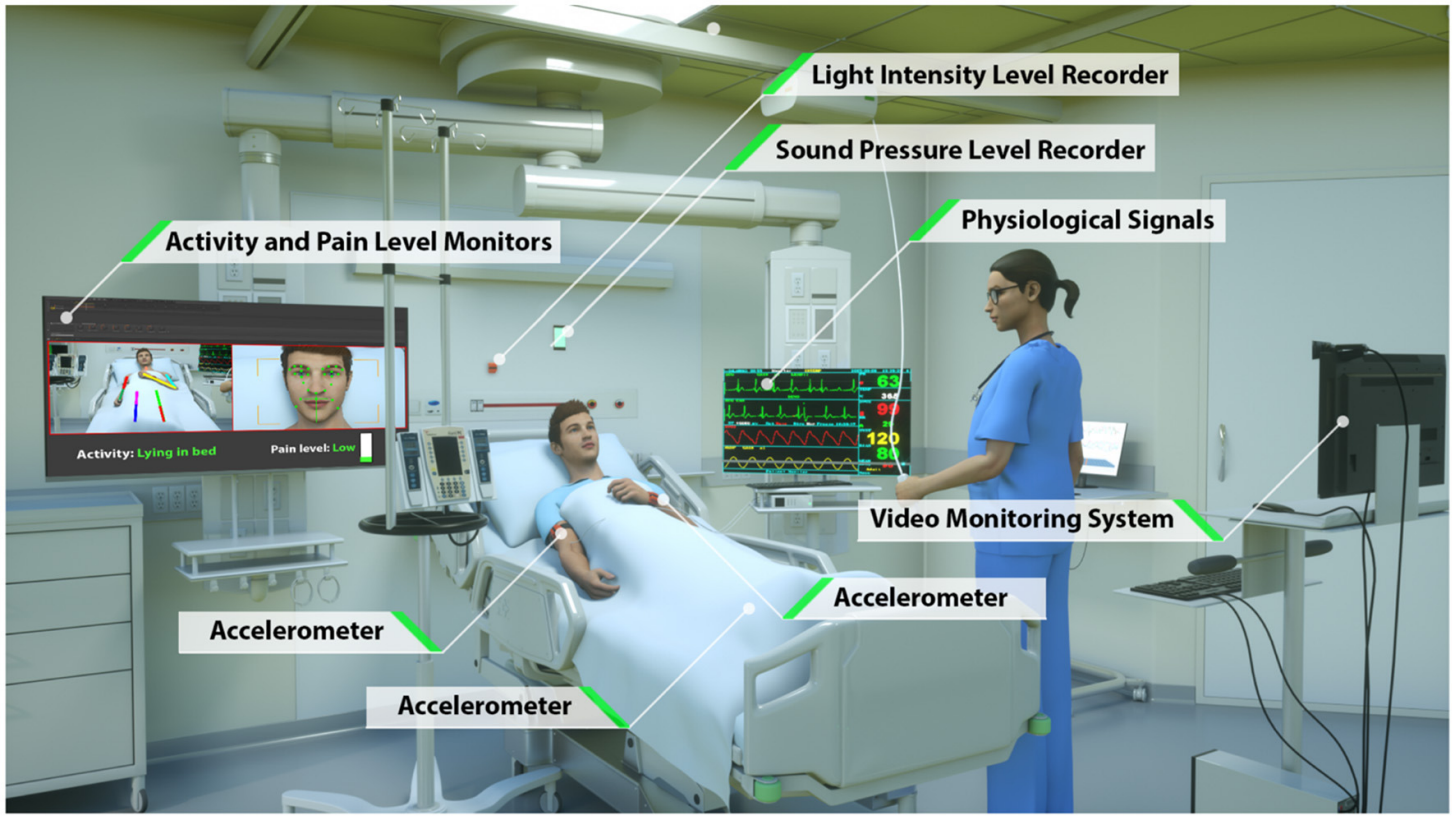
Intelligent ICU room introduced by Davoudi et al. (26). In this study, we augment traditional vital signs from electronic health records with novel activity data from wrist-worn accelerometer sensors.

To overcome the limitations of current approaches to automated patient monitoring, recent studies have begun to explore the benefits of intensive care units augmented with continuous and pervasive sensing technology. In a study dubbed the Intelligent ICU, Davoudi et al. augmented traditional EHR-based data with patient-worn accelerometer sensors, room-equipped light and sound sensors, and a patient-facing camera (26) (Figure 1). Their initial pilot study demonstrated the positive impact of these novel clinical data streams in characterizing delirium in a small prospective cohort of ICU patients. While these non-traditional ICU data sources have shown promise for improving modeling of critically ill patients, Intelligent ICU rooms equipped with pervasive sensors are still in early stages of research.

In this study, we build upon the work of Davoudi et al. by utilizing the data generated by Intelligent ICUs for automated patient acuity assessment using deep learning techniques. In particular, we show that by augmenting existing EHR data with continuous activity measurements via wrist-worn accelerometer sensors, models are better able to capture illness severity by way of more accurate predictions of hospital discharge disposition. We overcome the issue of small sample size in the Intelligent ICU cohorts by employing transfer learning techniques, where learned knowledge and representations from a much larger cohort of EHR-only patients is used as a starting point for subsequent incorporation of the non-traditional data streams. By combining transfer learning with augmented ICU monitoring, our work demonstrates the utility, efficacy, and future promise for using Intelligent ICUs for more personalized and accurate illness severity assessments.

## 2. Materials and Methods

### 2.1. Study Aims

The primary goal of our study is to characterize the effectiveness of augmenting traditional EHR patient data with a novel Intelligent ICU data source as it pertains to patient acuity assessment using machine learning techniques. Specifically, we combine datasets consisting of several common vital signs with continuous measurements from a wrist-worn activity sensor, and use these augmented datasets to make predictions of a patient's eventual successful or unsuccessful hospital discharge as a proxy for illness severity. In this study, we consider a discharge to home or rehabilitation facility as successful, with in-hospital mortality or transfer to another hospital or hospice being considered unsuccessful.

Our second aim is the evaluation of transfer learning as a solution to cope with the issue of small sample size in our prospective Intelligent ICU patient cohort. We hypothesized that building upon algorithmic patient representations from a much larger cohort of traditional ICU stays would result in improved predictive performance in the smaller cohort of interest.

### 2.2. Study Cohorts

Our primary cohort of interest, which we refer to as the Intelligent ICU cohort, includes 51 distinct ICU admissions at University of Florida Health between September 2015 and February 2020. These intensive care episodes were made up of 51 unique patients undergoing 51 unique hospital encounters, and occurred within specialized intensive care units outfitted with several unconventional monitoring systems (Figure 1). The Intelligent ICU cohort included 33 successful discharges (64.7%) and 18 unsuccessful discharges (35.3%).

For transfer learning experiments, we constructed a much larger second cohort of 48,400 distinct ICU admissions occurring at University of Florida Health between January 2011 and July 2019. We refer to these admissions as the Conventional ICU cohort, as it comes from standard intensive care units that contain only the data available in typical EHR systems. These ICU admissions included 32,184 patients undergoing 45,147 unique hospital encounters. The Conventional ICU cohort included 36,392 successful discharges (75.2%) and 12,008 unsuccessful discharges (24.8%).

This study was approved by University of Florida Institutional Review Board by IRB 201900546. A summary and comparison of admission and demographic descriptors for each cohort is shown in Table 1.

**Table 1:** Summary of Intelligent ICU and Conventional ICU cohorts.

**Descriptor**	**Intelligent ICU**	**Conventional ICU**
	**(*n* = 51)**	**(*n* = 48,400)**
Patients, *n*	51	32,184
Hospital encounters, *n*	51	45,147
Hospital length of stay (days), median (25th, 75th)	14.9 (9.0, 21.7)	7.3 (4.2, 12.9)
Successful hospital discharge, *n* (%)	33 (64.7)	36,392 (75.2)
Unsuccessful hospital discharge, *n* (%)	18 (35.3)	12,008 (24.8)
ICU stays, *n*	51	48,400
ICU length of stay (days), median (25th, 75th)	10.3 (6.4, 13.9)	3.0 (1.6, 6.0)
Age (years), median (25th, 75th)	63.2 (43.3, 73.0)	61.2 (48.8, 70.9)
Body mass index, median (25th, 75th)	27.3 (22.9, 33.2)	27.1 (23.1, 32.1)
Charlson comorbidity index, median (25th, 75th)	2.0 (0.0, 4.0)	2.0 (0.0, 4.0)
Sex		
Female, *n* (%)	18 (35.3)	20,188 (44.7)
Male, *n* (%)	33 (64.7)	24,959 (55.3)
Race		
White, *n* (%)	44 (86.3)	34,702 (76.9)
Black, *n* (%)	5 (9.8)	7,615 (16.9)
Other, *n* (%)	2 (3.9)	2,830 (6.2)
Ethnicity		
Hispanic, *n* (%)	1 (2.0)	1,677 (3.8)
Not Hispanic, *n* (%)	50 (98.0)	42,989 (96.2)
Language		
English, *n* (%)	51 (100.0)	44,396 (98.3)
Non-English, *n* (%)	0 (0.0)	751 (1.7)
Marital status		
Married, *n* (%)	24 (55.8)	20,513 (48.3)
Single, *n* (%)	14 (32.6)	13,606 (32.1)
Divorced, *n* (%)	2 (4.7)	4,149 (9.8)
Widowed, *n* (%)	1 (2.3)	3,341 (7.9)
Separated, *n* (%)	2 (4.7)	545 (1.3)
Life partner, *n* (%)	0 (0.0)	292 (0.7)
Provider		
Medicare, *n* (%)	27 (57.5)	23,203 (53.9)
Private insurance, *n* (%)	13 (27.7)	10,707 (24.9)
Medicaid, *n* (%)	4 (8.5)	6,612 (15.4)
Uninsured, *n* (%)	3 (6.4)	2,550 (5.9)
Smoking status		
Smoker, *n* (%)	7 (15.6)	8,514 (21.1)
Former smoker, *n* (%)	21 (46.7)	15,779 (39.1)
Never smoker, *n* (%)	17 (37.8)	16,060 (39.8)

### 2.3. Data Extraction and Processing

#### 2.3.1. Traditional EHR Data

Whether receiving care in an Intelligent ICU or conventional ICU room, all patients have the same set of data recorded into their electronic health records. In this study, for both cohorts we extracted all ICU measurements of six commonly recorded vital signs: diastolic blood pressure, systolic blood pressure, heart rate, respiratory rate, oxygen saturation (SpO2), and temperature.

A multivariate time series of vital signs was constructed for each ICU stay by temporally ordering measurements and resampling to a fixed 1-h frequency, where the mean value was taken if multiple measurements existed in the same 1-h window. We extracted measurements from the entirety of each ICU stay, thus each vital sign sequence was variable length based on the number of hours a patient was in the ICU.

#### 2.3.2. Intelligent ICU Data

The novel environmental and pervasive sensing technology was unique to the 51 ICU stays occurring in our Intelligent ICU cohort. Among all available non-traditional data sources (Figure 1), in this pilot study we opted to explore the added utility of wrist-worn activity sensors. Since this is the first study of its kind, we intentionally chose to limit the inclusion of novel data sources as a starting point for exploring and discussing the potential benefits of Intelligent ICU rooms for enhanced patient acuity assessment. While, we provide a brief summary of the technology and data streams contained within Intelligent ICUs, we refer interested readers to the work of Davoudi et al. (26) for a more comprehensive overview.

Patient activity data was collected from an Actigraph GT3X sensor (ActiGraph, LLC. Pensacola, Florida) placed on the patient's dominant wrist when possible, and on the opposite wrist when medical devices prevented ideal placement. These sensors generate activity based on magnitude of wrist motion (27) and sample at a frequency of 100 Hz. In this study, we aggregated accelerometer data into 24-h intervals, and extracted nine statistical features from each consecutive 24-h window after ICU admission. These features included minimum, maximum, mean, variance, standard deviation, immobile count, interquartile range (IQR), root mean square of successive differences (RMSSD), and standard deviation of RMSSD.

A summary of all features used in our models for both the Intelligent ICU and Conventional ICU cohorts is shown in Table 2.

**Table 2:** Summary of features used in our experiments.

**Feature**	**Intelligent ICU**	**Conventional ICU**
	**(*n* = 51)**	**(*n* = 48,400)**
	**Median (25th, 75th)**	**Median (25th, 75th)**
Vital signs		
Diastolic blood pressure, mmHg	62.3 (53.0, 73.0)	63.0 (54.0, 73.0)
Systolic blood pressure, mmHg	123.0 (110.0, 139.0)	121.0 (107.0, 137.5)
Heart rate, beats/min	91.0 (80.0, 103.0)	85.0 (74.0, 97.0)
Respiratory rate, breaths/min	18.3 (15.0, 22.5)	18.0 (15.0, 21.0)
Oxygen saturation (SpO2), %	98.0 (95.0, 100.0)	97.0 (95.0, 99.0)
Temperature, °C	37.0 (36.7, 37.5)	36.9 (36.7, 37.3)
Wrist activity, action counts		
Minimum	0.0 (0.0, 0.0)	N/A
Maximum	62.7 (39.5, 97.1)	N/A
Mean	2.9 (1.2, 6.3)	N/A
Variance	58.6 (16.3, 135.5)	N/A
Standard deviation	7.7 (4.0, 11.6)	N/A
IQR	1.8 (0.0, 8.0)	N/A
RMSSD	7.1 (4.2, 11.6)	N/A
RMSSD standard deviation	1.0 (0.9, 1.1)	N/A
Number immobile	0.6 (0.4, 0.8)	N/A

#### 2.3.3. Final Data Preprocessing

For both sequences of patient data, outliers were capped at the 1st and 99th percentiles, with cutoff points determined by the development set of each individual experiment. Any missing extracted feature values in the resulting sequences were imputed with the previous sequence value, if it existed, otherwise with the feature median based on each experiment's development set. Finally, each feature was standardized to zero mean and unit variance based on values from the development set of each experiment.

### 2.4. Models

**Figure 2:**
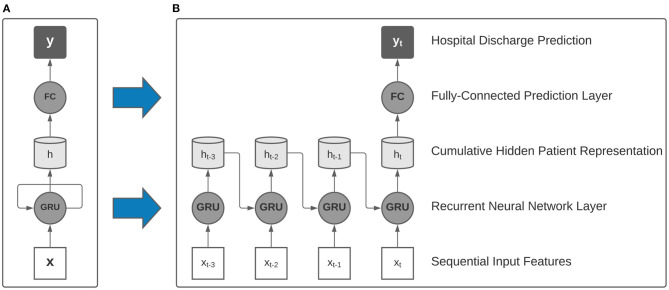
Collapsed **(A)** and expanded **(B)** view of a single-layer recurrent neural network with gated recurrent units (GRU) used as building blocks in our model. After processing multi-resolution sequences of vital signs and activity sensor data, a final hospital discharge prediction was made using the final hidden representation.

In this study, we employ single-layer recurrent neural networks (RNN), a class of deep learning algorithms that are well-suited to processing sequential data and have been validated in literature as accurate clinical models for patient acuity assessment (13). In particular, our RNN models utilize gated recurrent units (GRU) and a linear prediction layer that is used to make a discharge prediction after processing each 24-h data window (Figure 2). As each sequential window's features are made available, the model learns a real-time cumulative representation of patient state that is used to predict patient illness severity.

Our study involved the training of two distinct families of recurrent neural networks that were designed to handle either only traditional ICU data, or traditional data augmented with the multi-modal Intelligent ICU data. When using the augmented dataset of both EHR and Intelligent ICU data, we utilized a parallel RNN architecture comprised of two recurrent neural networks that independently processed each data source on separate time scales, with the concatenation of hidden representations passed to the linear prediction layer for assessing final predicted hospital discharge status.

### 2.5. Experiments

**Figure 3:**
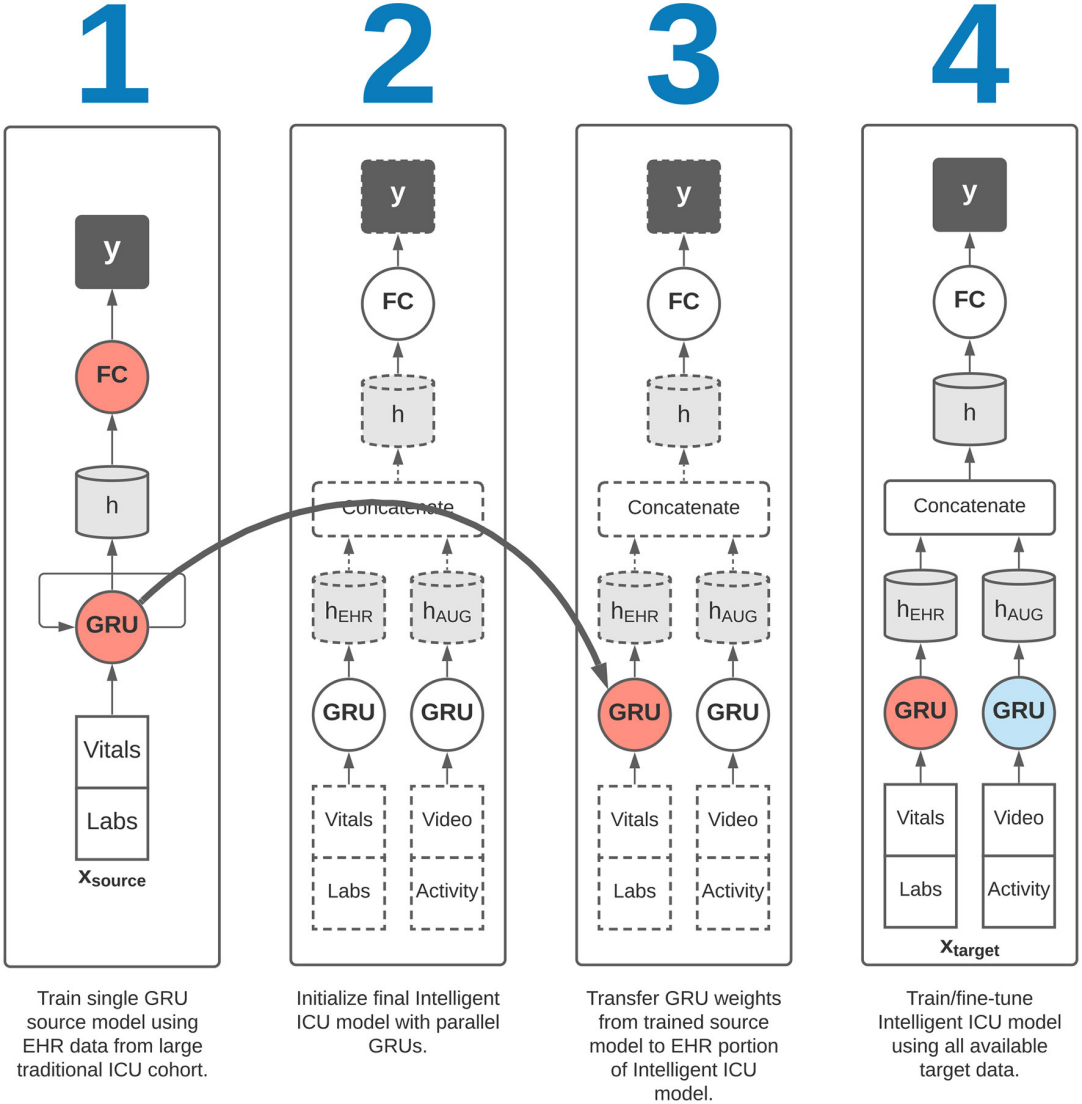
Overview of transfer learning procedure. Our final Intelligent ICU model incorporates pre-trained representational knowledge from a source deep learning model trained only on electronic health records data from a large cohort of traditional ICU stays (*n* = 48,400).

Corresponding to our aims in section 2.1, we sought to evaluate the effectiveness of augmenting traditional EHR data with Intelligent ICU data for making predictions of eventual successful or unsuccessful hospital discharge in our cohort of patients undergoing care in Intelligent ICU rooms. Given the small sample size of our Intelligent ICU cohort (*n* = 51), we also sought to explore the potential benefits of applying the technique of transfer learning, whereby a source model, typically trained on a larger dataset, is used to initialize a smaller model that is subsequently fine-tuned on the smaller dataset of interest. In our transfer learning experiments, we first trained a recurrent neural network on the Conventional ICU cohort (*n* = 48,400), and transferred its internal RNN weights and biases to a separate model for predicting illness severity in the Intelligent ICU cohort. This transfer learning process is shown in Figure 3.

This study includes four experimental variants designed to evaluate our study aims, all using the same discharge disposition targets. All results are reported on the target cohort of 51 ICU encounters occurring in Intelligent ICU rooms.

First, we sought to evaluate predictive performance in the target cohort without the application of transfer learning. The first of these experiments involved the training of a single RNN model on only the EHR data available in the target cohort. Next, we performed a similar experiment using a parallel RNN model with both the EHR and Intelligent ICU data available in the target cohort. These two experimental settings were designed to characterize potential benefits of augmenting traditional EHR data with more novel Intelligent ICU data streams.

We then repeated the above two experiments in conjunction with a transfer learning procedure. In each of these two transfer learning experiments, we first trained a single RNN model on the EHR data from the large Conventional ICU cohort of 48,400 ICU stays. Upon completion of training this source model, we initialized the RNN weights and biases in the EHR portion of the Intelligent ICU models using the final trained RNN weights and biases from the Conventional ICU models (Figure 3). The Intelligent ICU models were then trained as normal using the data available in the target Intelligent ICU cohort, in a process known in transfer learning literature as fine-tuning. In both transfer learning experiments, only the final RNN designed to process EHR data was initialized with pre-trained weights, as the Conventional ICU cohort did not contain any novel data sources. Consequently, the final RNN for processing the novel data sources was always trained starting with randomly initialized values.

All experiments on the target Intelligent ICU cohort were performed using 100 repeated trials of randomized five-fold cross-validation stratified by discharge target labels. Within each of the 100 cross-validation experiments, we retained the mean area under the receiver operating characteristic curve (AUROC) across all five validation set folds. 95% confidence intervals were obtained based on percentiles from these 100 averaged AUROC results. When training the large source model on the Conventional ICU cohort, we used the final chronological 20% of ICU stays as validation data, and obtain confidence intervals via 100 bootstrapped iterations based on validation set predictions.

When training a deep learning model, we used a random 20% of the development set for early stopping. Our deep learning models used hidden units of 128 dimensions across all layers, and were trained in batches of 32 samples with an Adam optimizer with learning rate 10^−3^ and *L*2 weight decay of 10^−3^. All layers used 25% dropout.

## 3. Results

Training the single RNN model on 80% of the large Conventional ICU cohort and evaluating on the remaining 20% validation set resulted in an AUROC of 0.752 (95% CI: 0.743–0.763). This trained model was used in all later transfer learning experiments, where the recurrent weights and biases were transferred to the final Intelligent ICU model as shown in Figure 3.

The single-cohort and single-RNN Intelligent ICU model using EHR data alone resulted in an AUROC of 0.734 (95% CI: 0.622–0.830). Augmenting the input data with both novel Intelligent ICU data sources and combining with the parallel RNN model resulted in an AUROC of 0.743 (95% CI: 0.644–0.842).

After the application of transfer learning using the model trained on the Conventional ICU cohort, the single-RNN model using only EHR data from the Intelligent ICU cohort resulted in an AUROC of 0.828 (95% CI: 0.557–0.951). The transfer learning model using the augmented dataset of EHR and Intelligent ICU data sources resulted in an AUROC of 0.915 (95% CI: 0.772–0.975).

Results for all experimental settings are summarized in Table 3.

**Table 3:** Hospital discharge prediction results for all experimental settings.

**Target cohort**	**Input data**	**Training scheme**	**AUROC (95% CI)**
Conventional ICU	EHR data	Single cohort	0.752 (0.743–0.763)
Intelligent ICU	EHR data	Single cohort	0.734 (0.622–0.830)
Intelligent ICU	EHR + Intelligent data	Single cohort	0.743 (0.644–0.842)
Intelligent ICU	EHR data	Transfer learning	0.828 (0.557–0.951)
Intelligent ICU	EHR + Intelligent data	Transfer learning	0.915 (0.772–0.975)

## 4. Discussion

In this study, we have provided the first attempts at incorporating cutting-edge pervasive sensing technology for patient monitoring and precise acuity assessments in the intensive care unit. Based on data from the Intelligent ICU environment of Davoudi et al. (26), we explored the performance impact of augmenting deep learning models with two novel data streams for the prediction of successful vs. unsuccessful hospital discharge as a measure of patient illness severity.

Several important takeaways can be gleaned from the performance results summarized in Table 3. When comparing single-cohort models trained on EHR data alone, the model trained on the larger Conventional ICU cohort of 48,400 ICU stays relatively outperformed a similar model trained on the much smaller Intelligent ICU cohort of 51 ICU stays (AUROC: 0.752 [95% CI: 0.743–0.763] vs. 0.734 [95% CI: 0.622–0.830]). While not unexpected given the large disparity in cohort sample sizes, the relatively small magnitude of difference between the cohorts is an interesting outcome, as one might expect an even larger discrepancy in model accuracy. While potentially attributable to a variety of factors, these results might suggest clear input patterns associated with improving or worsening health condition that yield diminishing returns as the sample size exponentially increases.

Given the results in Table 3, it is also clear that augmenting traditional EHR data with novel activity features in our single-cohort Intelligent ICU model marginally improved its predictive performance (AUROC: 0.743 [95% CI: 0.644–0.842] vs. 0.734 [95% CI: 0.622–0.830]).

Model accuracy was greatly improved using both input dataset variants after the application of transfer learning. When considering EHR data alone, transfer learning increased model accuracy from an AUROC of 0.734 (95% CI: 0.622–0.830) to an AUROC of 0.828 (95% CI: 0.557–0.951). Compared with the results yielded by the single-cohort model in the large Conventional ICU cohort (AUROC: 0.752 [95% CI: 0.743–0.763]), the final accuracy of the Intelligent ICU cohort was much higher. We speculate that these performance improvements point to the power of proper weight initialization in deep learning models, especially for clinical applications using relatively small patient cohorts. We note that although transfer learning with EHR data alone resulted in substantial gains in model accuracy over the model trained on the large Conventional ICU cohort, the prediction confidence interval in the small Intelligent ICU cohort was much wider (95% CI: 0.557–0.951 vs. 0.743–0.763), highlighting the large variability among the cross-validation repetitions. We speculate that this instability was due to the small size of the prediction cohort (*n* = 51). Given that this is a pilot study demonstrating transfer learning feasibility, we place less emphasis on the fact that absolute accuracy in the smaller cohort was greater than in the larger Conventional ICU cohort, which we partially attribute to sample size disparities. Instead, we focus on the relative performance increase in the same Intelligent ICU cohort, which clearly show the benefits of transfer learning in clinical situations where samples are not readily available.

Maximum overall performance was achieved when combining traditional EHR data with the novel Intelligent ICU data and a transfer learning approach (AUROC: 0.915 [95% CI: 0.772–0.975]). These results indicate the utility of augmenting traditional EHR data with pervasive sensing, and suggest that further research and incorporation of even more novel data streams could be beneficial to the real-time acuity estimation of critically ill patients. These results indicate the power of applying transfer learning in clinical settings with small patient cohorts. It was only when using transfer learning that the predictive benefits of augmented patient data truly became apparent. Similar to the experiments using only EHR data, we focus on the relative performance increase compared with the same augmented dataset in the Intelligent ICU cohort, which show clear benefits for using transfer learning to properly initialize model weights corresponding to electronic health record data from a much larger cohort of conventional ICU patients.

In all experiments using our target Intelligent ICU cohort of 51 ICU stays, the wide AUROC confidence intervals underscore the large variability among the repeated applications of cross-validation. This was not unexpected given the very small size of the Intelligent ICU cohort, especially when used with complex deep learning model architectures. However, when averaged over 100 repeated cross-validation trials, a more clear picture begins to emerge: predictive power is increased both when augmenting traditional vital signs with activity data, and when applying transfer learning, with optimal results achieved after implementing both techniques. We present these results as a pilot study indicating the feasibility of applying transfer learning to small cohorts of patients monitored with non-traditional data streams. While the small sample size of our target Intelligent ICU cohort is less than ideal, we speculate that relative performance increases within the same cohort show future promise for more extensive studies once more Intelligent ICU data becomes available.

Intelligent ICU rooms such as those used in our study are unfortunately rare in practice. However, we feel that pervasive sensing could play an important role in developing a more comprehensive and personalized representation of patient health, and we expect additional types of novel patient monitoring to become more common in future automated patient monitoring applications. Our preliminary results in predicting successful or unsuccessful hospital discharge using a subset of available Intelligent ICU data streams demonstrate the power of non-traditional patient data. As these novel clinical environments become more prevalent, our results also show the necessity of transfer learning approaches to jump-start models using these small augmented cohorts.

Non-traditional patient monitoring data that is not routinely measured in electronic health records as part of a typical hospital encounter provides a unique opportunity for enhancing clinical decision-making. As we have shown, the accuracy of automated methods for assessing illness severity can be improved when considering such types of novel sensing data. As pervasive sensing becomes more common in traditional intensive care settings, modern machine learning approaches can begin to better understand inherent patterns of data such as patient activity, facial expressions, environmental factors, and more. Augmented patient data can improve clinical decisions such as allocation of clinical resources, altering the characteristics of the ICU room environment, and can help provide objective measures of a patient's affective state and activity that can better inform clinical caregivers regarding appropriateness of medications or procedures.

This study was limited by the use of data from a single institution. Additionally, only a subset of EHR data and Intelligent ICU data was used in this preliminary study. Future work will incorporate all available novel sensing and EHR data, and will focus on even more granular illness severity estimations using higher frequency sensor measurements without aggregation to generate predictions on hourly or sub-hourly time scales. As temporal deep learning techniques continue to evolve, we believe their application to a wide array of both conventional EHR and sensor-based patient health data will lead to large improvements in clinical decision-making and patient outcomes as health trajectories become more accurately predicted and monitored using a more complete perspective on patient health.

## Data Availability Statement

The data analyzed in this study is subject to the following licenses/restrictions: UFHealth cohort data are available from the University of Florida Institutional Data Access/Ethics Committee for researchers who meet the criteria for access to confidential data and may require additional IRB approval. Requests to access these datasets should be directed to https://www.ctsi.ufl.edu/about/research-initiatives/integrated-data-repository/.

## Ethics Statement

The studies involving human participants were reviewed and approved by University of Florida Institutional Data Access/Ethics Committee.

## Author Contributions

BS, AD, TO-B, MR, and AB had full access to the data in the study and take responsibility for the integrity of the data and the accuracy of the data analysis. Study design performed by BS, AD, and PR. Conception of and data collection from the Intelligent ICU performed by AD, TO-B, AB, and PR. Analyses were performed by BS. Manuscript was drafted by BS and PR. Study supervision was performed by PR and AB. All authors contributed to the acquisition, analysis, and interpretation of data. All authors contributed to critical revision of the manuscript for important intellectual content.

## Conflict of Interest

The authors declare that the research was conducted in the absence of any commercial or financial relationships that could be construed as a potential conflict of interest.
